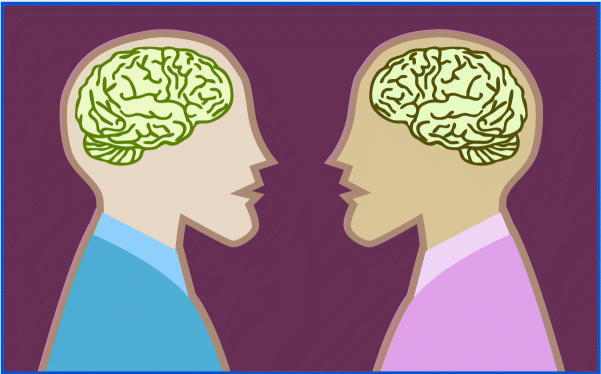# Headliners: Metal Toxicity: Lead Exposure May Affect Language Ability

**Published:** 2007-02

**Authors:** Jerry Phelps

Yuan W, Holland SK, Cecil KM, Dietrich KN, Wessel SD, Altaye M, et al. 2006. The impact of early childhood lead exposure on brain organization: a functional magnetic resonance imaging study of language function. Pediatrics 118:971–977.

Lead exposure is known to cause behavioral problems and learning deficits in children that persist into adulthood. Delays and/or deficits in intellectual ability, academic achievement, and psychomotor development have all been associated with childhood lead exposure. Localizing functional changes in the brain has been limited, however. Now NIEHS grantee Bruce Lanphear of the Cincinnati Children’s Environmental Health Center and colleagues report evidence of reorganization of the language centers of the brains of young adults with a history of childhood lead exposure, suggesting that lead exposure affects language ability.

The investigators recruited project participants from the Cincinnati Lead Study, an ongoing epidemiologic investigation into the long-term effects of childhood lead exposure that recruited pregnant women from 1979 to 1984. The subjects have been followed from birth and have had extensive documentation of lead exposure, medical history, neuromotor function, and academic achievement. Forty-two young adults participated in the current study on language ability. Their average childhood blood lead level was 14.2 μg/dL.

The Cincinnati team conducted functional magnetic resonance imaging on the subjects while they performed a “verb generation task.” Subjects were instructed to silently think of verbs in response to a noun. For example, if the noun “ball” was presented, the subject might think of verbs such as “throw,” “kick,” or “hit.” Accounting for significant potential confounders, the researchers conducted multivariable regression analysis to test the significance between brain language activation and mean childhood blood lead levels.

Higher mean childhood blood lead levels were associated with significant diminished activity in regions of the left hemisphere of the brain known to be responsible for language ability, along with compensation in regions in the right hemisphere. The authors note, however, that the compensatory alternative pathway does not necessarily yield performance equivalent to that achieved through normal brain pathway function. Similar adaptations have been documented in response to tumors, epilepsy, and stroke, though the effects seen in this study were not as severe as those observed with stroke.

## Figures and Tables

**Figure:**